# Liniment for Sore Nose

**Published:** 1881-01

**Authors:** 


Liniment for Sore Nose.—Hager recommends, for the soreness caused by acrid secretions, the following:

| | | |
|---|---|---|
| R. | Corrosive sublimate............ | 1-12 grain. |
| | Benzoic acid.................... | $\frac{3}{4}$ " |
| | Rose water..................... | 2 drachms. |
| | Diluted alcohol................. | $\frac{1}{2}$ " |
| | Glycerine....................... | $\frac{1}{2}$ " |
| | Tincture of opium............. | 10 drops. |

Apply three times a day with a camel's-hair brush.—*Ohio Med. Recorder.*

